# Performance of Baited Underwater Video: Does It Underestimate Abundance at High Population Densities?

**DOI:** 10.1371/journal.pone.0127559

**Published:** 2015-05-26

**Authors:** Ben Stobart, David Díaz, Federico Álvarez, Cristina Alonso, Sandra Mallol, Raquel Goñi

**Affiliations:** 1 Instituto Español de Oceanografía, Centro Oceanográfico de Baleares, Muelle de Poniente s/n, 07015, Palma de Mallorca, Spain; 2 South Australian Research and Development Institute, Port Lincoln Marine Science Centre, PO Box 1511, Port Lincoln, SA 5606, Australia; 3 Institut de Ciències del Mar, Consejo Superior de Investigaciones Científicas, Passeig Maritim de la Barceloneta, 37–49, 08003, Barcelona, Spain; 4 I.M.E.M. Ramón Margalef, Universidad de Alicante, Alicante, Spain; University of Waikato (National Institute of Water and Atmospheric Research), NEW ZEALAND

## Abstract

Video survey techniques are now commonly used to estimate animal abundance under the assumption that estimates relate to true abundance, a key property needed to make video a valid survey tool. Using the spiny lobster *Palinurus elephas* as our model organism, we evaluate the effectiveness of baited underwater video (BUV) for estimating abundance in areas with widely different population density. We test three BUV abundance metrics and compare the results with an independently obtained abundance index from trammel-net surveys (Trammel). Video metrics used to estimate relative abundance include a value for total number of individuals per recording (TotN), the traditional maximum number of fish observed in a single video frame (MaxN), and the recently suggested alternative, the average of the mean MaxN from 5-minute periods throughout the duration of the recording (MeanN). This is the first video study of a wild population to include an estimate for TotN. Comparison of TotN with the other two BUV relative abundance metrics demonstrates that both of the latter lack resolution at high population densities. In spite of this, the three BUV metrics tested, as well as the independent estimate Trammel, distinguished high density areas from low density areas. Thus they could all be used to identify areas of differing population density, but MaxN and MeanN would not be appropriate metrics for studies aimed at documenting increases in abundance, such as those conducted to assess marine protected area effectiveness, as they are prone to sampling saturation. We also demonstrate that time of first arrival (T1) is highly correlated with all of the abundance indices; suggesting T1 may be a potentially useful index of abundance. However, these relationships require further investigation as our data suggests T1 may not adequately represent lobster abundance in areas of high density.

## Introduction

The use of video to study marine life has increased over the past twenty years, and a variety of video survey techniques are now commonly used for sampling marine populations (see reviews by [1–2]). Amongst others, the advantages of using video include the removal of the time and depth limitations associated with diver surveys, the potential for reductions in survey costs, the ability to check images as many times as necessary and the relative ease of training observers to process recordings. Importantly video sampling techniques are also non-extractive and therefore well suited for studies on marine protected areas [3–4]. While video techniques do not necessarily outperform traditional sampling techniques such as visual census [5], they are free from diver bias and in many cases they have been demonstrated to perform better (e.g. [6–8]). In recent years the use of video systems has increased as technological improvements have made them cheaper and easier to use. Improvements include better video quality, increased filming times, a reduction in the size and cost of video recorders and changes to the recording media from tapes to direct storage on hard drives [2].

While video has been used for underwater surveys in many different ways [1–2], video systems are commonly used as “traps”, with bait added to attract species of interest to the camera field of view (e.g. [4, 7, 9–11]). Baited underwater video (BUV) relies on target species being attracted to a bait and has been demonstrated to provide better statistical power than un-baited systems in the detection of spatial and temporal changes to both the relative abundance of species and the structure of fish assemblages [12–13]. The latter is possible because BUV systems attract more species and sample higher species diversity than un-baited systems [1]. The main disadvantage of using BUV is the unknown area of attraction of the bait that can vary depending on the current [14] and estimating the attraction area to allow standardization of abundance estimates is inherently complicated (e.g. [15–16]). In addition, there may be variation in counts associated with interactions within and between species [17] and the upper limit of individuals that can be counted in the field of view which is normally space-limited [18].

The introduction of video hardware to sample marine populations required the development of methods to retrieve information from recordings, particularly metrics that could be used as a proxy for true abundance. The most commonly used metric for this purpose has been MaxN, generally defined as the maximum number of fish observed in a single video frame [9–10, 19]. MaxN is popular as it is relatively easy to obtain and provides the minimum number of individuals known to occur in a recording, ensuring individuals are not counted more than once, and is considered a conservative index of abundance. Thus, to date, this statistic has been used as the standard index of abundance for video estimates (e.g. [1, 8, 11, 17]). However, there is growing evidence that MaxN may provide dampened estimates of abundance with increasing true abundance, and therefore be prone to sampling saturation, as demonstrated by comparing MaxN with total numbers of fish in laboratory video deployments [20]. Thus using MaxN may result in positively biased indices of abundance for declining populations, or negatively biased indices when populations increase. If MaxN is prone to sampling saturation its use as a proxy for true abundance may be limited, in particular for studies that attempt to assess population recovery, such as those conducted in marine protected areas. Thus Schobernd et al. [20] proposed MeanCount, the mean number of fish observed in a series of snapshots over a viewing interval, as an alternative measure to MaxN because they found it to be linearly correlated with true abundance.

In this study we design, build and evaluate an effective, low cost, BUV system to sample the spiny lobster *Palinurus elephas* in and around the Columbretes Islands Marine Reserve (hereafter the “MPA”). We trialed BUV because the traditionally used method to capture this species for study is trammel netting which, while considered a highly efficient method for spiny lobster fishing [21–22], is an extractive method and therefore not ideal for use in a MPA. BUV offered the advantage of being non-extractive and lends itself well to sampling lobsters which are scavengers and therefore likely to be attracted to bait (e.g. [23]). Video is also ideal for use in the habitat depth range for *P*. *elephas* in and around the MPA of approximately 40–100 m, with possible extension to the full depth range of this species at other locations if required (200m; [24]). In this case underwater visual census by divers, the logical alternative non-extractive method, is not a viable option as the depth range lies below that considered safe to operate.

The aim of this study is to assess the performance of the BUV system and a range of metrics for measuring animal abundance in areas with widely different population density. We use the spiny lobster *P*. *elephas* as our model organism and set out to evaluate the effectiveness of our design for estimating lobster abundance using three different metrics: the traditional MaxN metric for relative abundance, the more conservative MeanN, and the total number of lobsters visiting the bait, TotN, a value for total abundance per recording. We were able to estimate TotN for this spiny lobster species by capitalising on a key feature of spiny lobsters, their unique body patterns, that allow the identification of individuals [25–26], allowing them to be tracked through the recording. We evaluated the relationship between MaxN, MeanN and TotN and compared them with independently estimated abundance metrics from trammel-net surveys. This is the first study for which a comparison of the conventional MaxN metric and a total per recording metric has been possible under natural conditions. We also investigate, for the first time, the relationship between abundance metrics and time of first arrival as a possible abundance indicator.

## Methods

### Ethics statement

No ethics permits were required for the described study, which complied with all the relevant regulations. Permission to work in the Columbretes Marine Protected (center at 39°52’32.70”N, 0°40’18.54”E) area was granted by the “Ministerio de Agricultura, Alimentación y Medio Ambiente” of Spain. The study did not use any endangered or protected species.

### Study area

**Fig 1:**
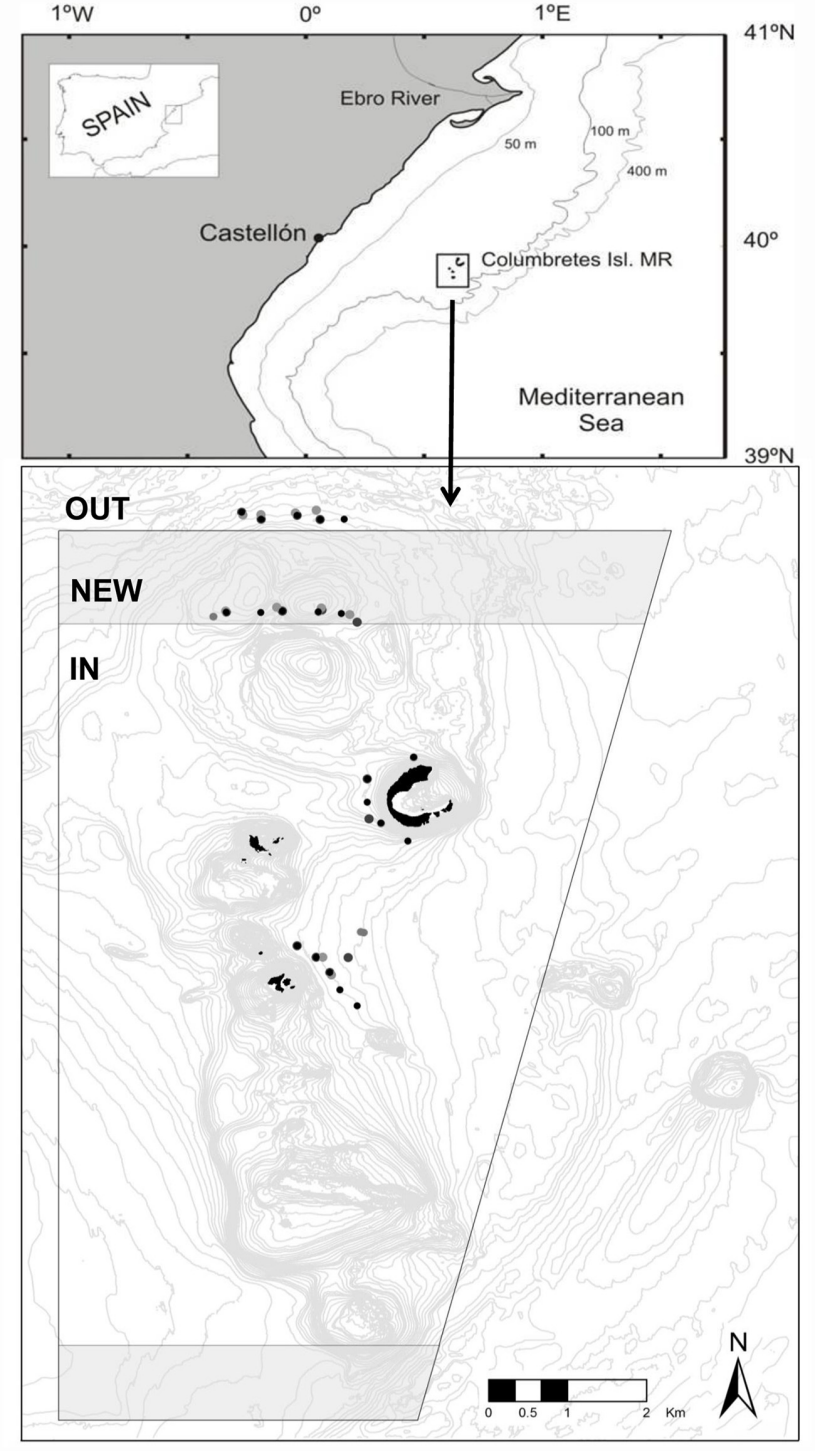
Map showing the location of the Columbretes Islands Marine Protected Area and detail of the levels of protection: IN, the MPA created in 1990; NEW, the area annexed to the MPA in 2009 and; OUT, the fished area outside the MPA. Marked points are the video deployment locations (on occasions shown as hollow points to facilitate viewing of multiple deployments in same general area).

This study took place in the Columbretes Islands Marine Reserve (MPA) and surrounding fishing grounds (Fig 1). The MPA is located 50 km east from the Mediterranean coast of Spain and protects 55 km^2^ of volcanic rock and coralligenous habitats (maërl beds) with patches of gravel, sand and mud that extend down to depths of ~80 m. Fishing grounds near the MPA (hereafter referred to as “OUT”) consist of patches of rock and maërl over expanses of gravel, sand and mud predominantly at depths of 60 to 80 m. The MPA was a traditional lobster fishing ground before it was closed to fishing in 1990. Since then, fishing effort in the region has been concentrated along the MPA boundaries and in nearby fishing grounds (<30 km from the MPA; [27]). The MPA was expanded in 2009 with a new area encompassing 7.43km^2^ (hereafter referred to as “NEW”). Legislation governing the MPA prohibits all fishing except very limited recreational (prior to 2002 recreational fishing was allowed) and commercial fishing trolling for pelagic species, which essentially renders the entire MPA a ‘no take zone’. Fishing regulations in the MPA are well enforced by a permanent ranger staff. Long-term monitoring of the lobster *P*. *elephas* conducted since 1998 indicate that density in the MPA is up to 20 times greater than in nearby fished grounds [28].

### Sampling design

This study was conducted at the MPA and adjacent fishing grounds. Data presented are from 45 video deployments conducted during summer months (August—September) between 2006 and 2012. Deployments were made inside the MPA (n = 22), within the newly created protection zone (NEW; n = 11) and in adjacent fishing grounds within 1 km of the MPA border (OUT; n = 12) (Fig 1). As an independent measure of abundance, we used data from experimental trammel net sampling (in the MPA and NEW zones), which has been the method of choice for monitoring lobster in the MPA since 1998, and by commercial trammel net fishing in the OUT area.

### Baited video unit

**Fig 2:**
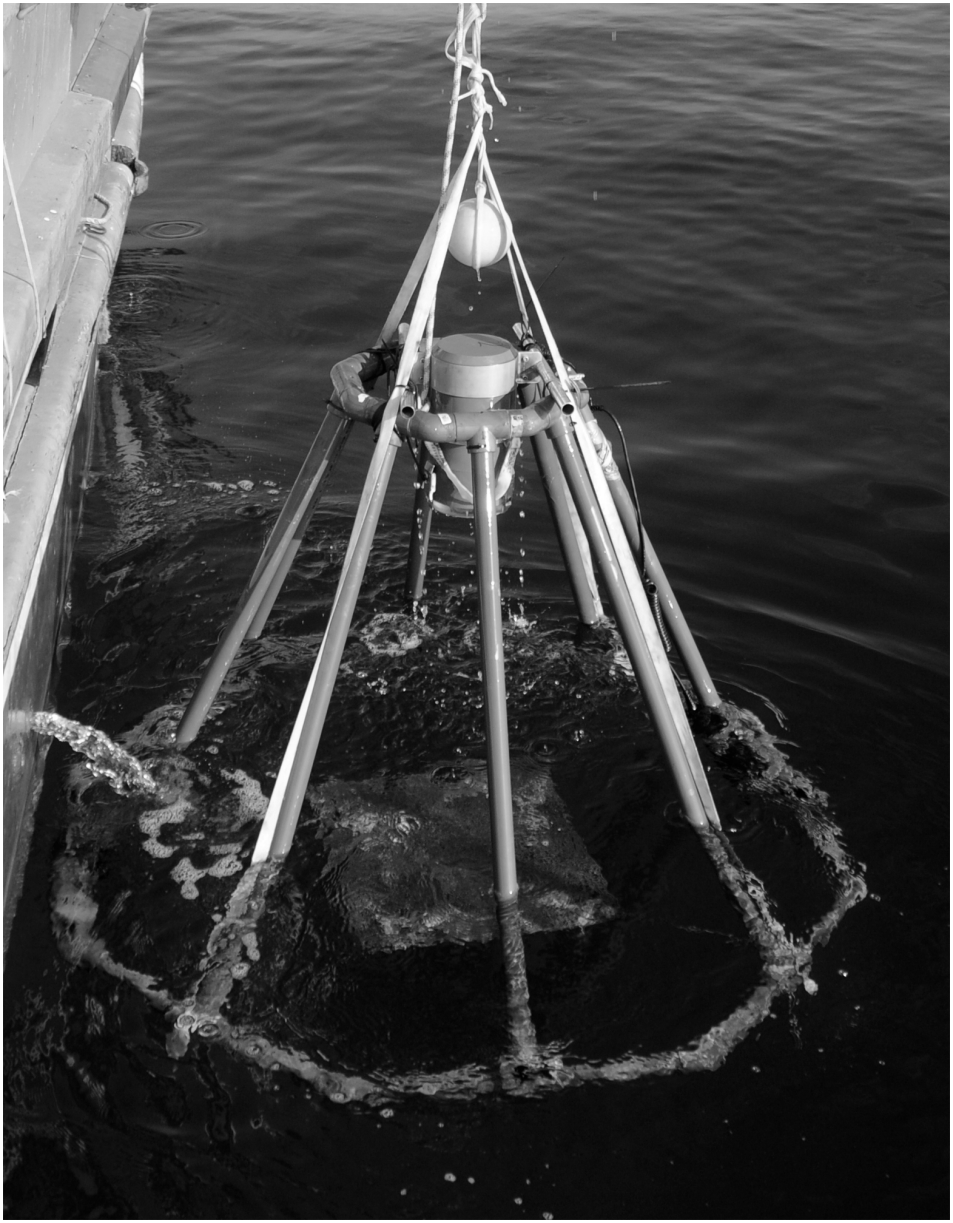
Baited underwater video system during deployment.

When considering BUV for sampling lobsters at depth we designed an economical, easy to build, BUV unit (similar to the fleet of BUVs designed by the Australian Institute of Marine Science; e.g. see [3]). Our design consisted of a digital video camcorder (Sony high resolution HDR-SR12E with BESEL super fish-eye 0.25 wide angle lens) held inside a custom made PVC underwater housing with acrylic viewing port. Recording time with a battery upgrade was in excess of 7 hours, with the duration of the light source used being the main limiting factor (approximately 5–7 hrs). The housing was mounted vertically on a PVC tubing octopod (40 mm tubing) and octagonal base (50 mm tubing; Fig 2). This structure has the advantage of being very strong and stable, and the PVC tubing can be put together with relatively inexpensive “off the shelf” joints to minimise manufacture costs. The octopod base was ballasted using chain threaded inside the tubing which reduced the risk of snagging and ensured the unit landed upright. Cameras provided a field of view of 90 x 60 cm, in the center of which was located a 60 x 40 cm bait bag. Recording was done on the “night shot” camera setting and illumination provided by a 10w lamp with red filter. The red filter was considered useful to reduce the chance of light interfering with lobster presence as lobsters react less to red light than white light [29].

Video units attached to a surface buoy were deployed just after dark and left overnight for retrieval early next morning. A secondary weight (50kg) was used to avoid current force on the surface buoy line dragging the unit during its deployment period. Prior to each deployment ~500g of crushed sardines (*Sardina pilchardus*), an oily bait commonly used in baited video studies [30], were placed inside the mesh bait bag.

### Video analysis

Video footage files were transferred to a hard drive for easy viewing on a PC. For each deployment trained operators then recorded the time of first appearance for each lobster (T1) and the maximum number of lobsters attending the bait in 5 minute time blocks over 5 hours. The maximum abundance of lobsters for each recording was then calculated using (1) the highest record for the maximum number of lobsters visible at any one point in the 5 minute time intervals (MaxN), (2) the mean number of lobsters (MeanN) which was the mean MaxN from 5-minute periods throughout the duration of the recording as calculated by Schobernd *et al*. [20] and, (3) the total number of lobsters appearing in the entire recording, with individual lobsters identified-in, and tracked through, the recordings using a combination of body patterns and/or damaged antennae and carapace size (TotN).

### Trammel net sampling

Experimental trammel net fishing surveys inside and around the MPA are conducted annually between June and September as part of a monitoring program which started in 1998 inside the MPA and in 2008 inside the NEW area. Trammel net abundance estimates from the fished area outside the MPA (OUT) come from onboard sampling of the commercial fishery around the MPA. Data used in this study were for the 2006 to 2012 period, totaling 135 fishing sets. The surveys are carried out with one of the commercial boats which operate in the area, with the same crew and gear type used in commercial fishing. Net length was 600 m and soak time 1 day for experimental fishing and 2–3 days for commercial fishing outside the MPA. As trammel net data was not available directly on the video deployment sites, we obtained an average trammel net abundance estimate for the vicinity of each video drop using trammel net data from the three nearest experimental trammel net deployments, all within an average of ~400m from the video sites (max 850m). By doing this, we hoped to account for much of the variability associated with the spatial distribution of lobsters within the vicinity of the video deployments. This allowed us to compare mean abundance estimates of lobsters obtained using trammel nets (heareafter referred to as “Trammel”) with those from video deployments. A detailed description of the trammel net survey methods is given in [31].

### Data analyses

Correlations between T1, MaxN, MeanN, TotN and Trammel were explored using standard regression techniques (log, polynomial, and linear). The relationship between time and MaxN was explored by obtaining the MaxN for each 5 minute time period and calculating its percentage in relation to the MaxN for the entire recording. Mean percent of MaxN ± SD was then computed across recordings and the relationship with time explored using a third order polynomial. Non-parametric Kendall’s tau (*τ*) correlations for pairwise comparisons was used to assess the correlation between abundance estimates made with the four methods (MaxN, MeanN, TotN and Trammel) and with the time of first arrival (T1) of video deployments. Significance level was adjusted using the Bonferroni correction for multiple comparisons (ɑ = 0.005).

Differences between the three areas sampled (IN, NEW and OUT) in estimates of T1 and lobster abundance (MaxN, MeanN, TotN and Trammel) were tested using non-parametric Kruskal-Wallis tests. Where differences were significant a post-hoc Dunn’s test was used to identify which areas were significantly different (Bonferroni correction ɑ = 0.017).

## Results

### Performance of lobster baited video

**Fig 3:**
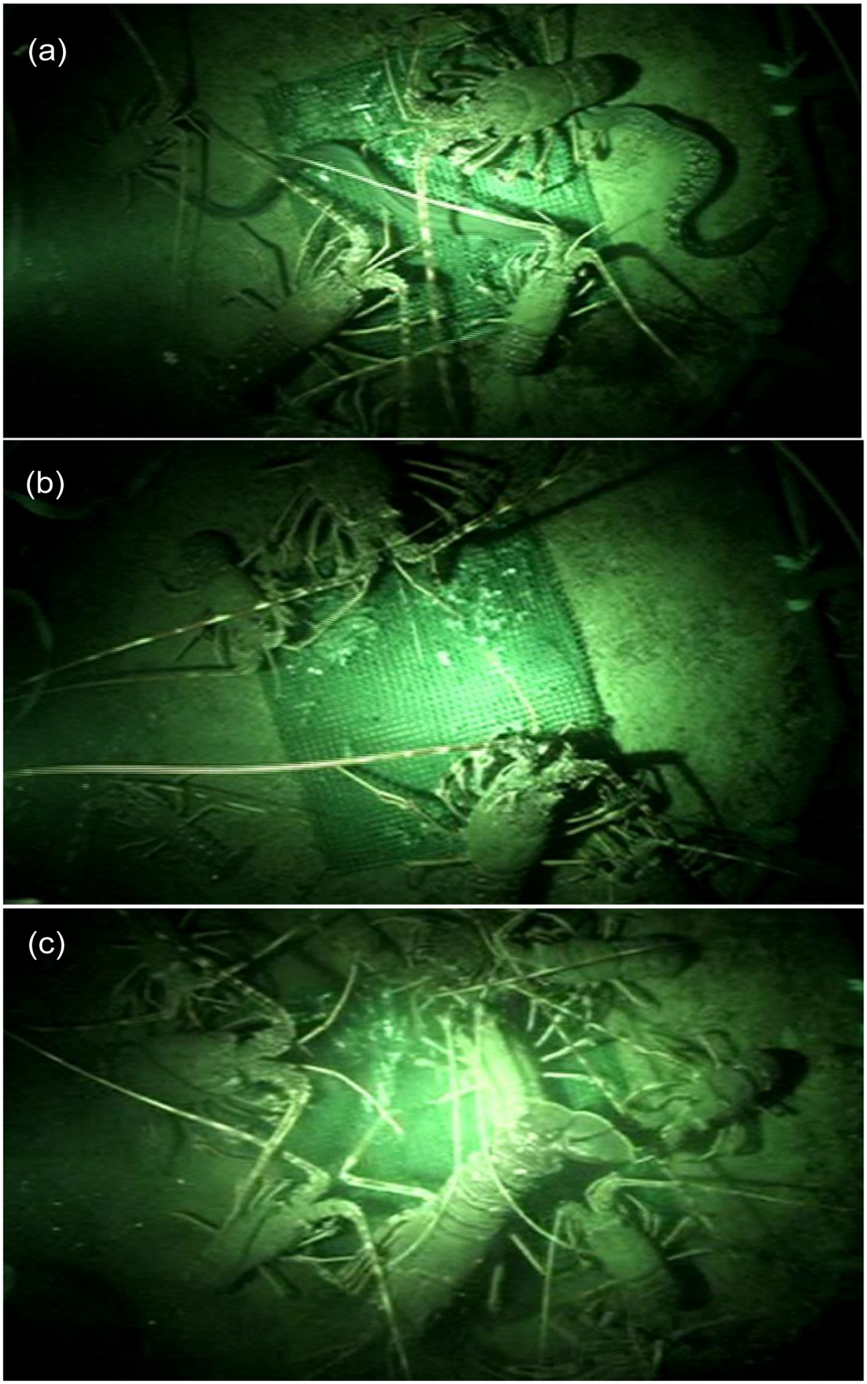
Video frame captures from the BUV system showing (a) lobsters (*P*. *elephas*) feeding at bait along with a moray eel (*M*. *helena*) and conger eel (*C*. *conger*); (b) large and small lobsters feeding at bait simultaneously; and (c) large number of lobsters feeding approaching the saturation point for the field of view.

The baited video system proved to be very reliable, landing upright on all occasions and with few failures to record. The main species attracted to the bait was the targeted species, *P*. *elephas* (Fig 3). In addition small unidentified crabs and hermit crabs were also relatively common. On rare occasions other species such as the eels *Conger conger* and *Muraena helena* (Fig 3a), grouper *Epinephelus marginatus*, forkbeard *Phycis phycis* and octopus *Octopus vulgaris* also attended the bait. With the exception of octopus leaving the bait when large eels arrived, there was no evidence of any one species excluding others from access to the bait. Within species, some large *P*. *elephas* were able to push smaller lobsters out of the way, but these still remained on the bait basket where it was common to have lobsters of all sizes side by side (Fig 3a–3c). Thus the presence of larger lobsters is not likely to have affected the counts.

**Fig 4:**
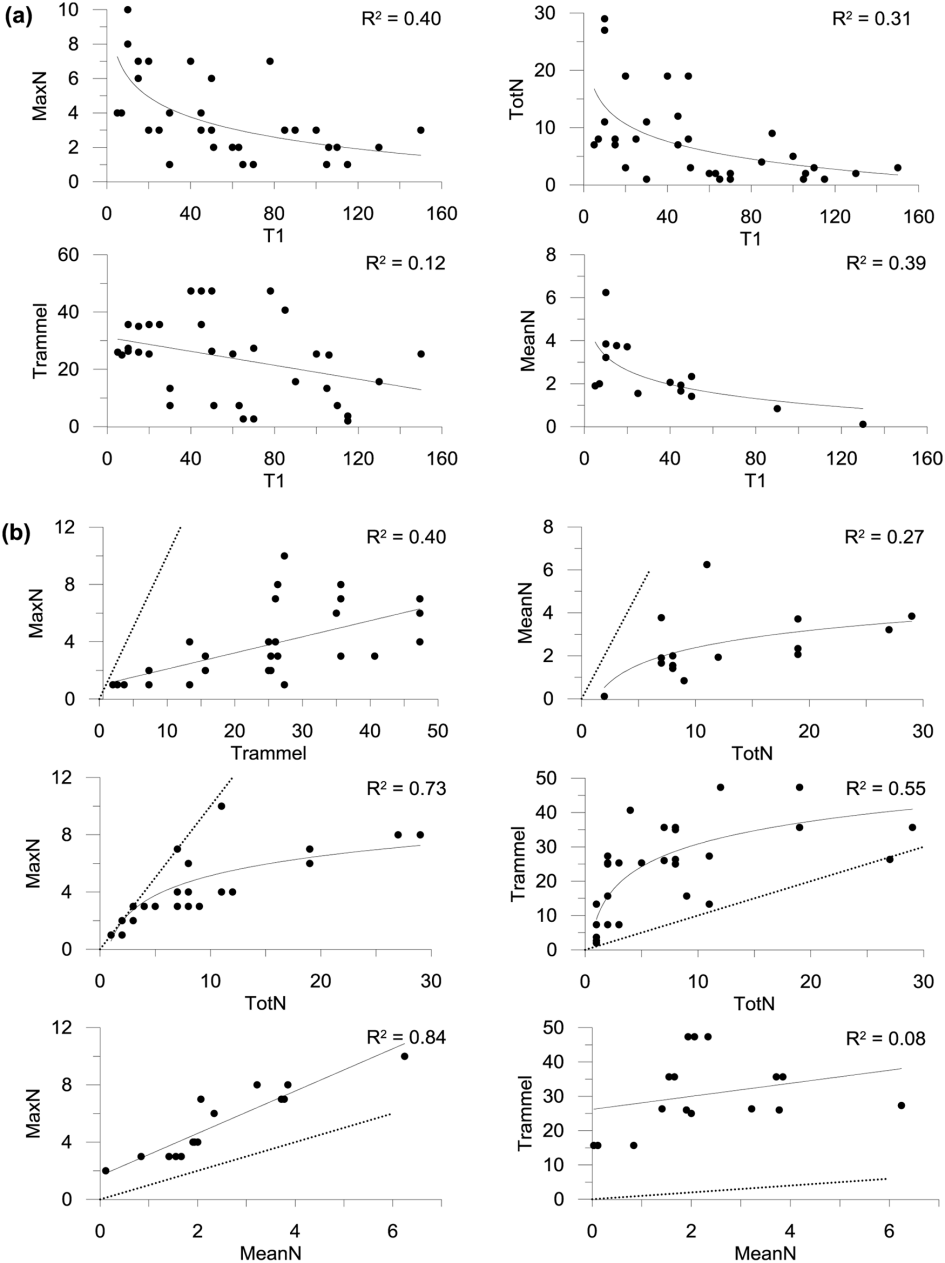
Relationships between (a) time of first arrival (T1) and lobster abundance metrics MaxN, MeanN, TotN, and Trammel; and (b) the estimation methods. Fitted solid lines are linear regressions and log fits (r^2^ shown on graphs). Dashed lines indicate equal values for both metrics.

**Fig 5:**
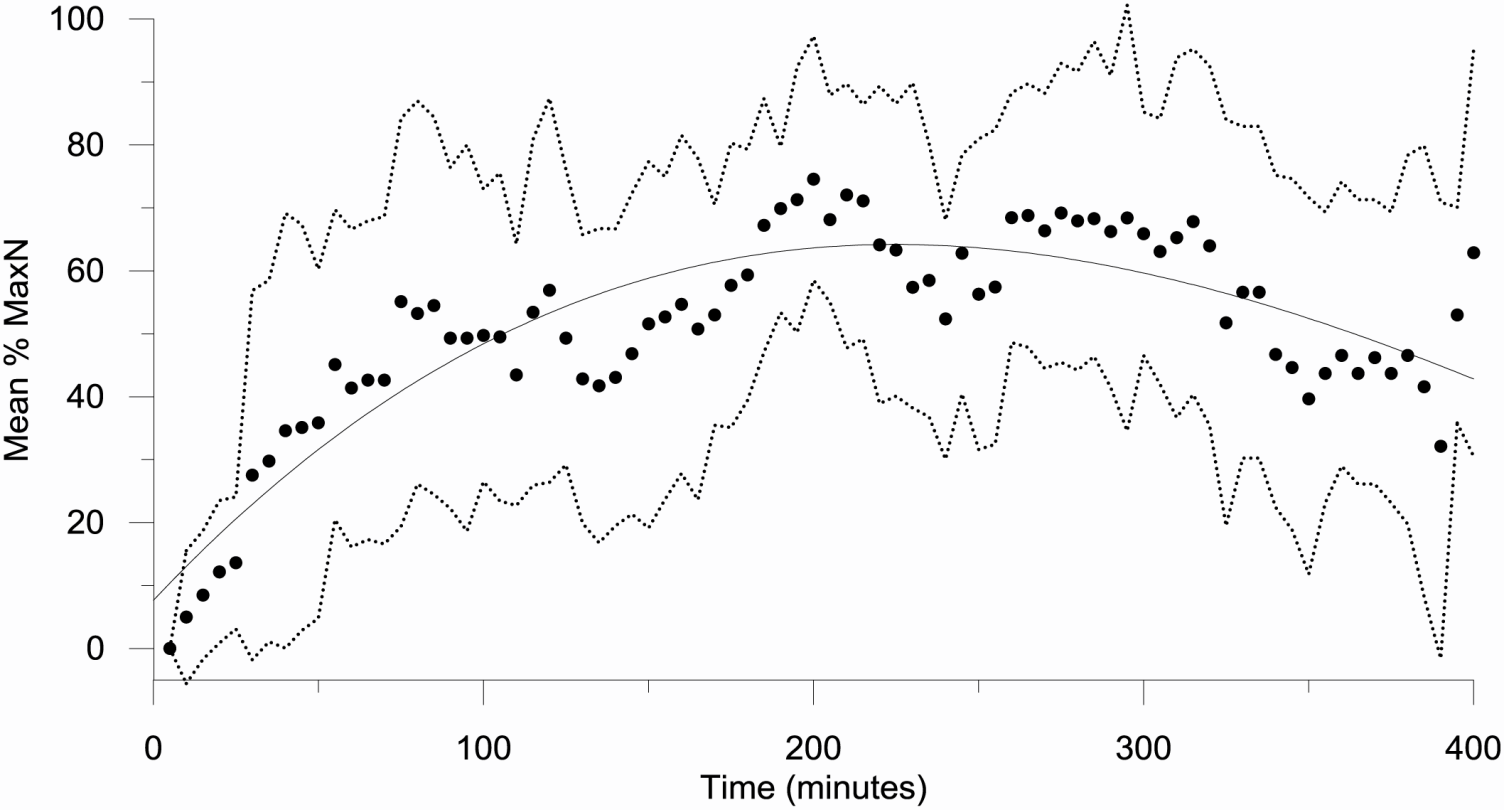
Mean percent of MaxN against video deployment time (minutes). Fitted solid line is a third order polynomial, dashed lines are the upper and lower standard deviations.

Time of first arrival (T1) varied from as little as 5 minutes up to 2:30 hrs and generally decreased with increasing MaxN on a logarithmic scale (Y = -1.724*Ln(X) + 10.269, r^2^ = 0.40; Fig 4a). Lobsters remained at the bait for an average of 66 minutes (±15 minutes SEM), with the maximum time a lobster remained at the bait being in excess of 5 hours. The highest MaxN reached was 10, with average values varying with protection level (see below). The relationship between mean percentage of MaxN and time was best explained by a third order polynomial where the percentage of MaxN generally increased fast over the first 1:30 hours and reached the highest mean percentage by 3:30 hours (Fig 5; Y = 7.653 + 0.556 * X—0.002(X,2) + 1.057E-006 * pow(X,3), r^2^ = 0.77).

The MaxN, MeanN and TotN estimates derived from the BUV recordings, and independently obtained Trammel estimates, were all highly correlated (Table 1; Fig 4b). All three estimates of abundance derived from the BUV were also highly negatively correlated with T1, while Trammel was not significantly correlated (Table 1; Fig 4a).

**Table 1:** Kendalls tau (*τ*) correlations between video MaxN, MeanN and TotN and Trammel estimates of lobster abundance obtained in the study.

	MeanN	TotN	Trammel	T1
MaxN	**0.87 (<0.001), n = 27**	**0.84 (<0.001), n = 34**	**0.59 (<0.001), n = 35**	**-0.51 (<0.001), n = 34**
MeanN		**0.74 (<0.001), n = 27**	**0.57 (<0.001), n = 27**	**-0.64 (<0.001), n = 27**
TotN			**0.59 (<0.001), n = 34**	**-0.45 (<0.001), n = 34**
Trammel				-0.27 (0.032), n = 34

Bonferroni corrected ɑ = 0.005. Significant differences are in bold.

**Fig 6:**
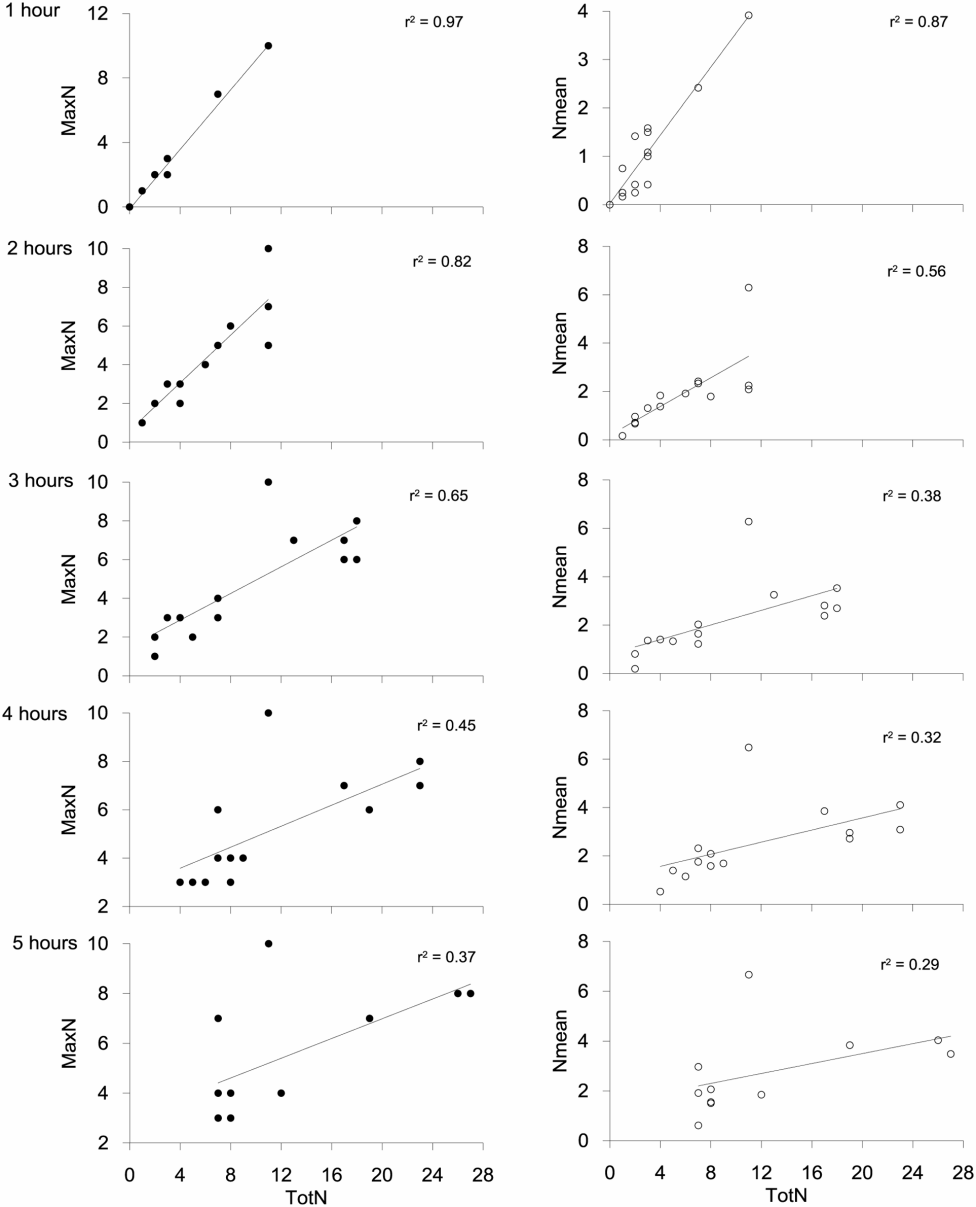
Relationships between MaxN and MeanN against TotN by deployment time. r^2^ and deployment times (hours) shown on graphs.

Both the highest MaxN (10) and MeanN (6) from all surveys were considerably lower than highest estimate of TotN (29; Fig 4b). MaxN was lower than TotN for ~60% of comparisons and, with the exception of one instance where MaxN and TotN were both 7, they were only equivalent when the total number of lobsters for the recording was 3 or less. MeanN is an average for the entire recording and thus, as expected, was lower than TotN in all cases. Importantly, the difference between TotN and both MaxN and MeanN was greatest in cases where TotN was high, as manifest by the levelling of the log curve for each of the comparisons (Fig 4b). Thus MaxN and MeanN are reaching a saturation point when the TotN exceeds approximately 10. This “saturation point” was only reached in the IN area, not in the NEW or OUT areas. The difference between MaxN, MeanN and TotN is not as apparent in the first two hours of recording, as suggested by the decreasing r values over time, probably due to the longer time needed for lobsters to accumulate at the bait (Fig 6). This suggests that longer deployment times are required to show the true extent of differences between areas of high and low density, as also suggested by the longer time taken (3:30 hrs) to reach Mean MaxN (Fig 5).

### Abundance estimates and T1 inside and outside the MPA

**Fig 7:**
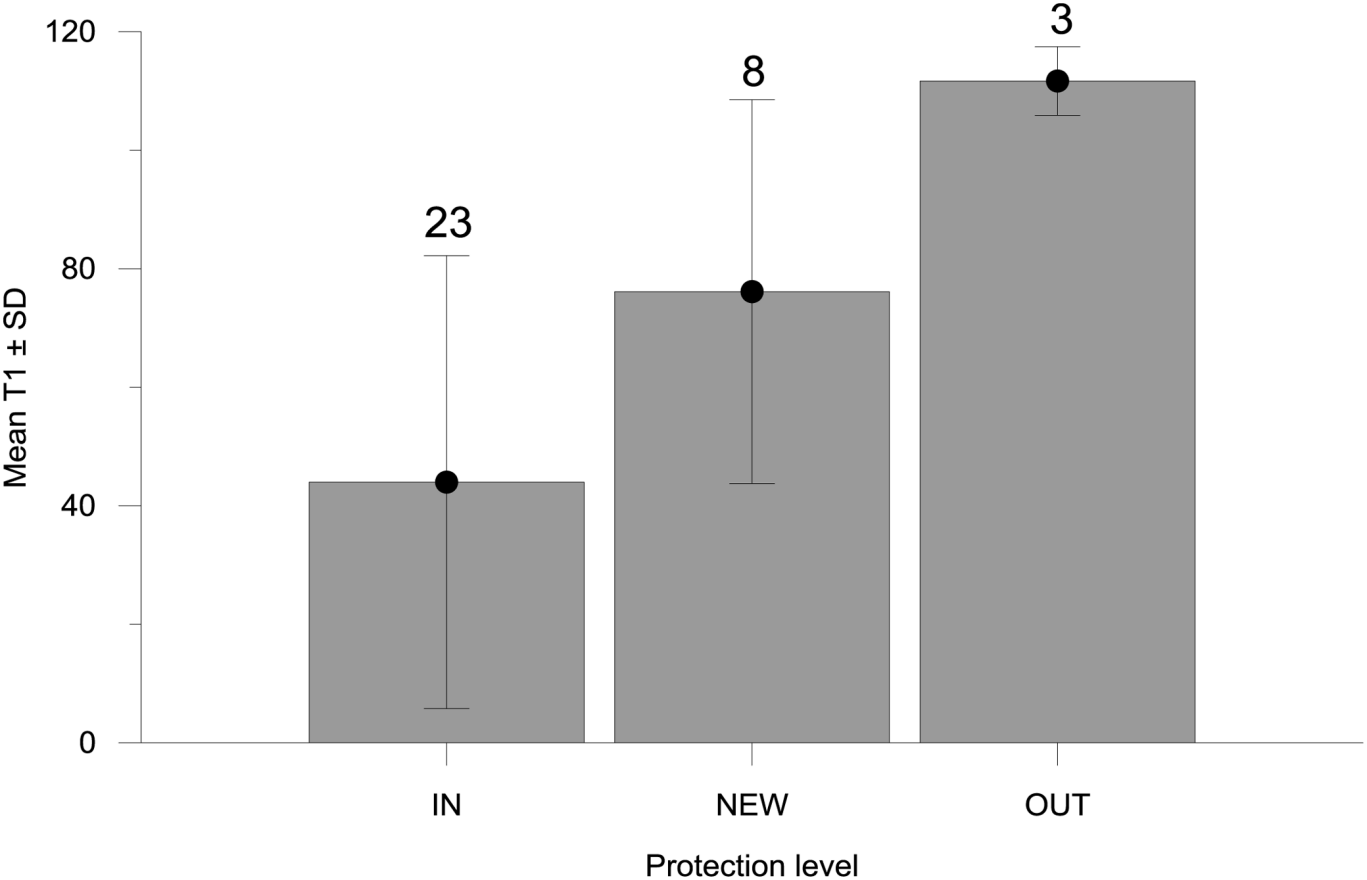
Mean time of first arrival ± SD by protection level (MPA, NEW and OUT). Numbers on top of error bars indicate sample size.

**Fig 8:**
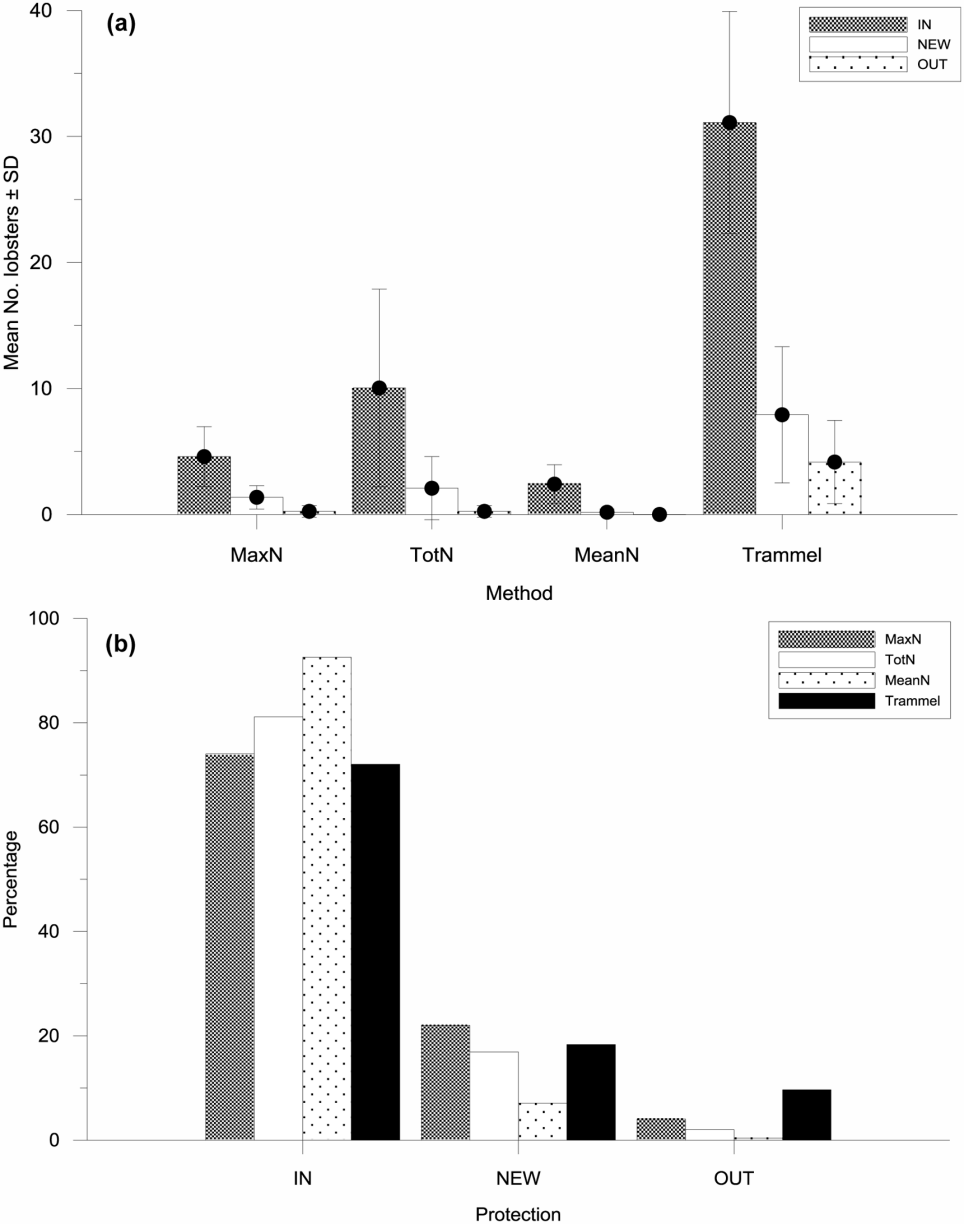
(a) Mean number of lobsters counted ± SD by protection level (IN, NEW and OUT; see legend) obtained from baited underwater video (MaxN, TotN and MeanN) and experimental trammel net fishing (Trammel). (b) Percent abundance comparison by method (see legend) and protection level (see legend).

Estimates of abundance obtained with BUV (all p ≤0.0001) and T1 (p = 0.007) differed between protection areas and are also consistent with those obtained for Trammel (p ≤0.0001). Mean T1 in videos inside the MPA was 42% lower than videos in the NEW area of the MPA and 65% lower than videos deployed outside the MPA, with T1 for IN significantly different from OUT, but not between other combinations (Table 2; Fig 7). This is clear indication that lobsters arrive faster at the bait inside the MPA than they do outside, and suggests T1 may have some application as a proxy for abundance. The reduced number of videos for estimating means of T1 for OUT was due the high number of video deployments outside the MPA (70%) that did not record any lobsters, reflecting the lower abundance outside the MPA. MaxN, MeanN, TotN and Trammel estimates of abundance were also significantly higher inside the MPA than OUT (Table 2; Fig 8a) and did not show a significant difference between NEW and OUT. However, in contrast with T1, they also showed a significant difference between IN and NEW. Importantly, while the abundance estimates from the four methods used were quite different from the different levels of protection, their percentage differences were similar between protection levels (Fig 8b). While the percentages were similar, it is noteworthy that estimates of lobster abundance from MeanN had a higher relative proportion at the IN protection level, possibly due to the influence “time at bait” has on the MeanN estimate. In contrast, trammel net estimates had a higher relative proportion outside the MPA, likely associated with the longer soak time, but also possibly the larger area fished by trammel nets.

**Table 2:** Calculated p-values for multiple pairwise comparisons of time of first arrival (T1) and the four methods used for estimating lobster abundance, against the three levels of protection (IN, NEW and OUT), using Dunn’s procedure.

	Protection level comparison		
Compared metric	IN/NEW	IN/OUT	NEW/OUT
T1	0.032	**0.007**	0.260
MaxN	**0.001**	**<0.0001**	0.113
TotN	**0.003**	**<0.0001**	0.073
MeanN	**0.002**	**<0.0001**	0.073
Trammel	**<0.001**	**<0.0001**	0.283

Bonferroni corrected ɑ = 0.017. Significant differences are in bold.

## Discussion

The BUV system tested proved to be a reliable method for estimating relative density of lobsters at depth and an effective, non-destructive, alternative to trammel net abundance estimation. We show, for the first time for a wild population, that the conventional BUV abundance metric MaxN, used as a measure of abundance for most BUV studies (e.g. [8, 11, 17]) lacks resolution at high population densities, such as those found in well protected MPAs [28, 32]. We demonstrate this by tracking individual lobsters throughout recordings, allowing us to estimate the total number of lobsters visiting the bait (TotN), and show that when lobster numbers are high, MaxN tends to asymptote. The point from which MaxN and TotN deviate is therefore likely the BUV field of view saturation point, lending weight to the concern that there is an upper limit to the number of target individuals that can occupy a BUV field of view [7, 18], be it due to a physical limit to the space within the field of view or the bait canister, constraints associated with species behavior or the declining probability of individuals all being within the frame of view simultaneously as abundance increases. This is consistent with a recent laboratory experiment by Schobernd et al. [20] that found MaxN was nonlinearly related to true abundance, and prone to sampling saturation. In addition, the other BUV metric we tested, MeanN, also showed a similar relationship with TotN and is therefore unlikely to be a more appropriate metric to use than MaxN. While Schobernd et al. [20] suggested MeanN may be useful as an alternative metric to MaxN for indexing abundance of fish, their trials may not have been close to the point of sampling saturation, and they concluded that further analysis would be appropriate before concluding that MeanN could be a viable substitute for MaxN. There is also evidence that TotN is not linearly related to Trammel, suggesting a limit to counts from BUV may have been reached. However, direct comparison of these two methods is complicated by the longer soak time and larger sampling area of Trammel.

In spite of the differences between the methods outlined above, they all demonstrated that the abundance of lobsters was significantly greater inside the MPA than outside and that the NEW area in the MPA established in 2009 has not yet reached the same density of lobsters as the IN area that has been established since 1990. Therefore, all of these relative measures of abundance can be used to assess differences due to protection level and, in this case, that protection leads to higher abundance. In addition, while the magnitude of the abundance estimates we found between methods differed considerably, they all revealed a similar proportional difference between the levels of protection. In particular, the corresponding patterns obtained for the independent metric Trammel and the BUV metrics highlight the potential for BUV to become a non-extractive alternative to Trammel. Other BUV studies, mostly focused on sampling fish, have also demonstrated that BUV is suitable for detecting both spatial (e.g. [7, 9]) and temporal differences [33] in relative abundance, while studies that have compared abundance indices obtained from BUV with those from alternative sampling methods have had mixed results. These ranged from a high correlation in estimates between methods (e.g. longline—[9, 33]; seine nets—[34]; fish traps—[6]), to clear differences between them (underwater visual census—[7]). Thus correspondence between BUV and other sampling methods depends on the species being evaluated and the method being compared (see review by Mallet and Pelletier [2]). Importantly, our findings also indicate that MaxN and MeanN would not be the metrics of choice to conduct a study aimed at following the recovery of a population after rebuilding or protection measures have been implemented, such as in an MPA, as they would likely asymptote prior to full recovery, leading to the premature conclusion that recovery was complete. Willis et al. [7] also noted that the upper limit for MaxN could be “problematic when attempting to detect differences between areas of high fish abundance”.

The close match highlighted above for the comparison of the proportions (percentages) obtained from the relative abundance metrics for the different levels of protection is reassuring. However, while they suggest all methods are suitable for detecting differences between levels of protection, there are differences that allude to the potential biases the different methods may have. In particular, MeanN gives a much higher percentage inside the MPA, and is very low outside. A likely explanation is that MeanN is influenced by the number of time blocks in which lobsters are present, and thus its value is raised inside the MPA. This bias, and the evidence that MeanN becomes saturated at high densities, suggests it may not be a suitable metric to substitute for MaxN as suggested by Schobernd et al. [20]. In contrast, Trammel is clearly more efficient at capturing lobsters at lower densities, in particular in the OUT area. In this case the likely cause of the bias is the longer soak time outside the MPA as well as the larger area being sampled by each Trammel deployment. It is noteworthy that despite this added “fishing capacity”, catches from Trammel are not greatly different from those estimated by TotN that is limited to the relatively small video field of view and a smaller soak time. This can be in part explained by the fact that many lobsters will not be trapped by the net, as has also been found using fish and lobster traps rigged with video cameras that showed higher filmed abundances than were caught [6,23]. In addition, the bait used for BUV is attracting lobsters from a wider area which may be considerable, as lobsters arrived at the bait up to 2.5hrs after deployment. However, the effective attraction distance is difficult to estimate as, amongst others, it will depend on the current speed and direction (e.g. [14]).

Using “off the shelf” components made building the BUV system relatively cheap and it was both easy to use and reliable. These are all features that were integral to the design brief and allow cost effective operations. In addition, recent changes to the cost, resolution and size of video hardware are already enabling us to design a cheaper and smaller version of our system with better image quality. The fact that the target species, *P*. *elephas* was the most frequently sampled visitor to the bait and abundance peaked within the recording period, demonstrates that the BUV system and methodology used is well suited to this species. Further benefits from the system include the ability to obtain reliable estimates of lobster size, determine the benthic habitat sampled and record species behavior. With the cost of the system decreasing, the long time taken to analyse video recordings remains a limitation to the use of this system that requires further attention. Processing time has been highlighted as one of the main limitations of video surveys [2–4], with the need to break up recording analysis into time increments in order to estimate MaxN adding to the “bottleneck” [3]. The highest mean percentage of MaxN was reached within 3:30 hrs, while the relationship between MaxN and TotN continues to deteriorate between four and five hours. This indicates that lobster BUV deployments should ideally last at least 3–4 hours and contrasts with suitable deployment times for fish which are generally regarded adequate at under an hour (e.g. [4, 10, 35]). Thus the added deployment time required for the lobster sampling ads to the processing time required, and therefore to the cost of analysing the video recordings. In comparison with MaxN, estimation of TotN was an even more time consuming process due to the complexity of tracking individual lobsters throughout the recording. However in this instance TotN, a metric that to our knowledge has until now not been obtained for marine species in the wild, is critical to evaluating the limitations of MaxN and thus the added effort is justified. In view of the time investment required, and the fact that many species may not have unique patterns/features that allow identification of individuals, it is not surprising that TotN has not been explored previously. However, given time and resources this metric could be estimated as many marine species subject to video analysis have useful body markings. For example, bony fishes [36–37] and sharks [38–39] often have body patterns specific to individuals that could be used to track individuals through recordings. Importantly, modern video imagery provides better resolution than was available in the past, allowing use of finer scale markings and, in addition, the markings used do not have to be stable in time beyond the duration of the recording.

Our data demonstrate that all three estimates of abundance derived from the BUV are highly negatively correlated with T1, suggesting that T1 may be a reliable proxy for abundance. As T1 can be quickly obtained from recordings, this relationship may be a useful way to dramatically reduce the processing costs associated with analyzing video recordings. However, at the high lobster abundance of the MPA reported here T1 was already close to zero, so it may not be as useful at higher abundance levels. In addition, T1 did not show the significant difference between the IN and NEW areas which was detected using the measures of relative abundance. This reduced sensitivity is likely due to the decrease in sample size for T1 that cannot be estimated when no lobsters are detected in the video recordings, whereas zero counts are represented in estimates of relative abundance. Correlation between time of first arrival and abundance has been reported previously ([34, 40]), although a study by Farnsworth et al. [15] using modelled data concluded that there was “no support from statistical theory” for their use. However, in view of the potentially large time savings to video processing, further exploration of the sensitivity and usefulness of this metric should be considered.

In conclusion, the BUV system we developed proved to be reliable for estimating the relative abundance of *P*. *elephas*, almost exclusively attracting this species. Body markings specific to individual lobsters allowed us to estimate TotN and compare this estimation of the total number of lobsters attracted to the BUV with the traditionally used and more recently suggested metrics MaxN and MeanN, respectively. Our findings demonstrate that all the metrics used (TotN, MaxN, MeanN and Trammel) are able to distinguish between high density areas and low density areas. However, neither MaxN nor MeanN would be appropriate metrics for studies that seek to document increases in abundance, as they are prone to saturation and therefore likely to prematurely indicate that peak abundance has been reached. While TotN was useful to explore the relationships between these metrics, tracking lobsters through recordings requires a lot of video analysis time, so TotN is unlikely to be used extensively in future studies of lobsters or other marine organisms without the aid of new software to automate the process. Time of first arrival may be a useful measure for some studies, but further work would be required to determine if it can be a useful index of abundance in areas of high density.
